# Manifestations and Management of Trimethoprim/Sulfamethoxazole–Resistant *Nocardia otitidiscaviarum* Infection

**DOI:** 10.3201/eid2906.221854

**Published:** 2023-06

**Authors:** Katherine Fu, Kyle White, Aliaksandr Ramaniuk, Vidya Kollu, Daniel Urbine

**Affiliations:** University of Florida College of Medicine, Gainesville, Florida, USA

**Keywords:** Nocardia, *Nocardia otitidiscaviarum*, trimethoprim/sulfamethoxazole, chronic obstructive pulmonary disease, cutaneous abscess, drug resistance, antimicrobial resistance, bacteria, United States

## Abstract

*Nocardia* can cause systemic infections with varying manifestations. Resistance patterns vary by species. We describe *N. otitidiscavarium* infection with pulmonary and cutaneous manifestations in a man in the United States. He received multidrug treatment that included trimethoprim/sulfamethoxazole but died. Our case highlights the need to treat with combination therapy until drug susceptibilities are known.

*Nocardia* *otitidiscaviarum* was first described in 1924 after being isolated from a guinea pig with ear disease (*1*). *N. otitidiscaviarum* infections account for ≈5.9% of all *Nocardia* infections (*2*,*3*). *Nocardia* can cause systemic infections with varying clinical signs. Predisposing factors are chronic lung disease, corticosteroid use, HIV infection, solid organ transplant, and solid organ malignancy (*4*). Patterns of resistance to antimicrobial therapies vary widely among *Nocardia* species. We present a case of a man with pulmonary and cutaneous manifestations of a trimethoprim/sulfamethoxazole (TMP/SMX)–resistant *N. otitidiscaviarum* infection.

The patent was a 73-year-old man in Gainesville, Florida, USA, who had severe chronic obstructive pulmonary disease, coronary artery disease, and congestive heart failure. In June 2020, he sought care for fever, productive cough, dyspnea on exertion, and a 30-pound weight loss over 4 months. Six months earlier, he had undergone computed tomography (CT) of the chest because of his smoking history and concern for unintentional weight loss. Scans revealed a 3.4-cm spiculated, cavitary, right upper lobe mass with associated precarinal and right hilar adenopathy. Subsequent positron emission tomography/CT showed a hypermetabolic wedge-shaped subpleural area of consolidation (5.0 × 4.1 cm) in the right upper lobe, consistent with pneumonia. Antimicrobial drugs were administered. Follow-up CT chest 3 months later showed progressive disease bilaterally in the upper lobes (Figure, panel A). The patient was then hospitalized and underwent bronchoscopy with bronchoalveolar lavage (BAL). We sent cultures for bacterial, including mycobacterial, and fungal testing. Pending results of the BAL gram stain and cultures, we empirically prescribed broad-spectrum antimicrobial drugs (i.e., vancomycin, cefepime, and metronidazole) for pneumonia, and the patient was discharged after clinical improvement. The BAL sample showed gram-positive branching rods (Figure, panel B), and *N. otitidiscaviarum* grew on culture. The patient was readmitted to the hospital.

**Figure:**
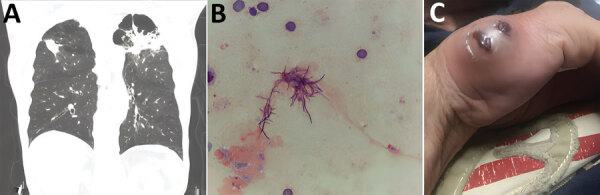
Pulmonary and cutaneous manifestations of *Nocardia otitidiscavarium* infection in a man in Gainesville, Florida, USA, 2020. A) Computed tomography of the chest showing progressive disease in the upper lobes**.** B) Bronchoalveolar lavage sample (Gram stain) showing gram-positive branching rods. C) Cutaneous abscess on the ulnar aspect of the right hand.

At readmission, examination was remarkable for distant heart and lung sounds and a prolonged expiratory phase. The patient had no noticeable skin lesions at that time. CT of the head with contrast (implantable cardioverter device was not compatible with magnetic resonance imaging) revealed no focal enhancing lesions. We empirically started intravenous TMP/SMX, intravenous amikacin, and oral levofloxacin. At the same time, the patient discovered a rapidly enlarging skin lesion along the ulnar aspect of the right hand (Figure, panel C). We incised and drained the 2.5-cm abscess, and *N. otitidiscaviarum* grew in cultures with broth microdilution. On the basis of sensitivity results (Table), we discontinued TMP/SMX and levofloxacin and added linezolid and clarithromycin; amikacin remained unchanged. Unfortunately, acute renal injury developed, and a new, prolonged QTc interval was seen on electrocardiogram. Those findings were attributed to either amikacin or clarithromycin, which prompted discontinuation of the aminoglycoside and macrolide. Repeat CT of the chest at week 6 of treatment showed disease progression. The patient subsequently decided to pursue palliative care and subsequently died. 

**Table:** Sensitivity results for *Nocardia otitidiscaviarum* isolated from a man in Gainesville, Florida, USA, 2020

Drug	Sensitivity
Amikacin	Sensitive
Amoxicillin/clavulanate	Resistant
Cefepime	Resistant
Ceftriaxone	Resistant
Ciprofloxacin	Intermediate
Clarithromycin	Sensitive
Doxycycline	Resistant
Imipenem	Intermediate
Linezolid	Sensitive
Minocycline	Intermediate
Moxifloxacin	Sensitive
Tobramycin	Intermediate
Trimethoprim/sulfamethoxazole	Resistant

Clinical manifestations of *N. otitidiscaviarum* infection may include pneumonia, brain abscess, lung abscess, skin abscess, muscle abscess, ocular infections, bacteremia, osteomyelitis, and septic joints (*4*). The risk factor for this patient was chronic obstructive pulmonary disease. The patient had pulmonary involvement and a rapidly developing skin abscess. 

Antimicrobial drug resistance of *N. otitidiscaviarum* is variable. The organism is usually sensitive to linezolid, amikacin, TMP/SMX, doxycycline, gentamicin, and minocycline. Susceptibility to carbapenems is limited; most activity is from imipenem compared with meropenem or ertapenem. *N. otitidiscaviarum* is generally resistant to amoxicillin/clavulanic acid, cefotaxime, ceftriaxone, ciprofloxacin, moxifloxacin, tobramycin, and clarithromycin (*3*–*5*). 

This case demonstrates the value of considering *Nocardia* infection for patients with cavitary lung disease and concomitant soft tissue abscesses. It also highlights the need to choose appropriate initial antimicrobial therapy. In the absence of a drug susceptibility profile, clinicians should approach the selection of antibiotics with a framework that considers disease severity, the epidemiologic probability of particular species, and the most likely resistance profiles of the species. Treatment can be narrowed after the species and susceptibilities are known.
